# fNIRS Exhibits Weak Tuning to Hand Movement Direction

**DOI:** 10.1371/journal.pone.0049266

**Published:** 2012-11-08

**Authors:** Stephan Waldert, Laura Tüshaus, Christoph P. Kaller, Ad Aertsen, Carsten Mehring

**Affiliations:** 1 Bernstein Center Freiburg, University of Freiburg, Faculty of Biology, Freiburg, Germany; 2 Institute of Biology I, University of Freiburg, Faculty of Biology, Freiburg, Germany; 3 Sobell Department of Motor Neuroscience and Movement Disorders, University College London, Institute of Neurology, London, United Kingdom; 4 Department of Neurology, University of Freiburg, University Medical Center Freiburg, Freiburg, Germany; 5 Institute of Biology III, University of Freiburg, Faculty of Biology, Freiburg, Germany; 6 Department of Bioengineering and Department of Electrical and Electronic Engineering, Imperial College London, London, United Kingdom; Universitat Politecnica de Catalunya, Spain

## Abstract

Functional near-infrared spectroscopy (fNIRS) has become an established tool to investigate brain function and is, due to its portability and resistance to electromagnetic noise, an interesting modality for brain-machine interfaces (BMIs). BMIs have been successfully realized using the decoding of movement kinematics from intra-cortical recordings in monkey and human. Recently, it has been shown that hemodynamic brain responses as measured by fMRI are modulated by the direction of hand movements. However, quantitative data on the decoding of movement direction from hemodynamic responses is still lacking and it remains unclear whether this can be achieved with fNIRS, which records signals at a lower spatial resolution but with the advantage of being portable. Here, we recorded brain activity with fNIRS above different cortical areas while subjects performed hand movements in two different directions. We found that hemodynamic signals in contralateral sensorimotor areas vary with the direction of movements, though only weakly. Using these signals, movement direction could be inferred on a single-trial basis with an accuracy of ∼65% on average across subjects. The temporal evolution of decoding accuracy resembled that of typical hemodynamic responses observed in motor experiments. Simultaneous recordings with a head tracking system showed that head movements, at least up to some extent, do not influence the decoding of fNIRS signals. Due to the low accuracy, fNIRS is not a viable alternative for BMIs utilizing decoding of movement direction. However, due to its relative resistance to head movements, it is promising for studies investigating brain activity during motor experiments.

## Introduction

Functional near-infrared spectroscopy (fNIRS) has recently attracted the interest of researchers working on motor control [1], [2] and brain-machine interfaces (BMIs; [3]). Unlike electroencephalography (EEG), fNIRS is not corrupted by electromagnetic noise and in contrast to functional magnetic resonance imaging (fMRI), fNIRS is portable.

Several studies have assessed the capabilities of fNIRS to investigate brain activity or as a potential control signal for BMIs [4]–[6]. Here, we investigated the characteristics of movement related fNIRS signals recorded above motor areas. So far, fNIRS signals have been shown to vary between rest and motor execution or imagery [7], [8] and to reflect motor task complexity [9] or force levels exerted in isometric hand/finger contractions [10]. fNIRS also allows to distinguish between left and right hand movements (performed or imagined), i.e. between left and right hemispheric motor activity [3], [11], [12].

Until now it has been unknown whether the spatial resolution and the signal-to-noise ratio of fNIRS are sufficient to investigate cortical activity related to different movements of the same limb. This question is of interest for motor control and BMIs, where the decoded direction of upper limb movements can be used as a control signal. Georgopoulos and colleagues [13] found a systematic dependence of single neuron spiking activity in monkey primary motor cortex on arm movement direction. Since then, systematic relations between spiking activity and various movement parameters have been found and online BMIs using decoded movement kinematics have been realized [14], [15]. Recently, researchers showed that not only spiking activity is tuned to movement parameters but also signals reflecting the activity of neuronal populations: local field potentials, electrocortico- (ECoG), magnetoencephalograms and EEG, see review [16]; corresponding online BMIs have been realized using ECoG [17] and MEG [18].

Research on respective tuning of hemodynamic signals commenced only recently. Studies using fMRI found single voxel activity to depend on hand/arm movement direction [19]–[21]. These studies suggest that the directional tuning of hemodynamic signals might be used as a BMI control signal. However, fMRI is not portable and therefore, not suitable for many BMI applications. fNIRS, a portable and low-cost technique, might instead be used to gather these signals but until now, it has remained unclear whether movement kinematics can be extract from fNIRS signals.

Therefore, we investigated fNIRS signals recorded simultaneously above several brain regions while subjects performed unilateral hand movements in different directions. We characterized the spatio-temporal properties of hemodynamic signals, quantified the strength of movement dependent differences and performed single-trial decoding of movement direction from fNIRS signals. Furthermore, we used a magnetic tracking system to measure and analyze the influence of head movements on fNIRS.

## Materials and Methods

### Subjects and Ethics Statement

Seventeen naïve subjects (aged between 20 and 45, average 26.8±5.6, seven female, ten male) participated in this study after giving written informed consent. From these subjects, five (aged between 22 and 30, average 26.6±2.8, one female, four male) participated in a pilot experiment and twelve (aged between 21 and 45, average 26.8±6.4, six female, six male) in the main experiment. The experimental procedures have been approved by the Ethics Committee of the University of Freiburg.

### Recording Systems – fNIRS

Hemodynamic brain activity was recorded using the fNIRS system ‘DYNOT-932’ from NirX Medical Technologies. The system operates at wavelengths of 760 and 830 nm and provides 32 optodes, from which 23 are detectors and nine are co-located sources. Flexible distribution of the optodes on the scalp was possible. Each detector measured the light intensity at 7.94 Hz sampling rate.

### Recording Systems - Head Tracking

**Figure 1:**
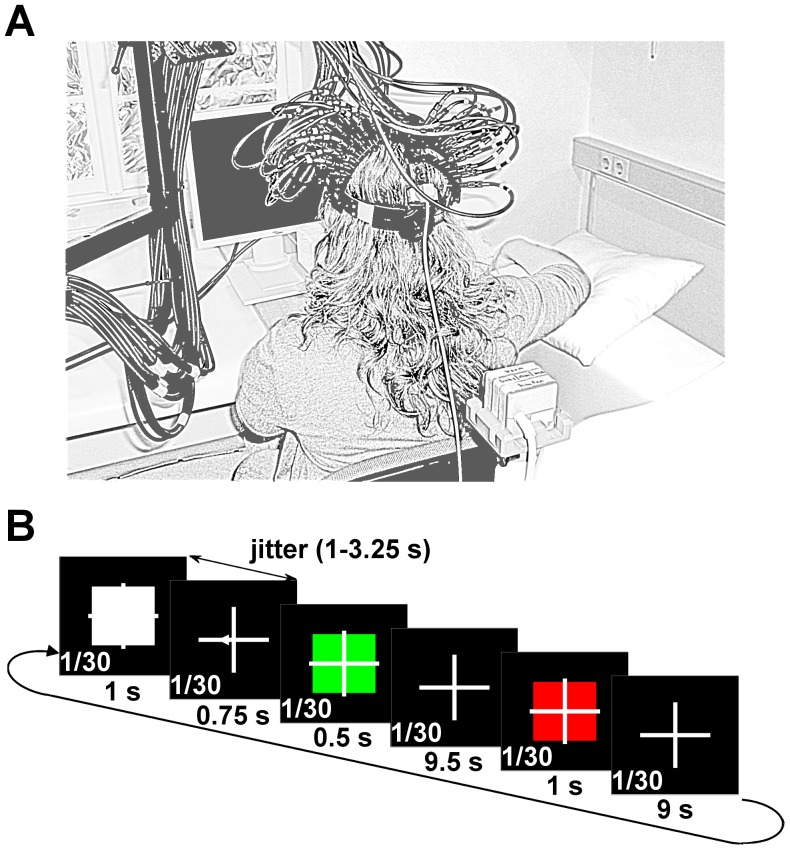
Experimental setup and trial structure. (**A**) Experimental setup showing the fNIRS optodes and the source and sensor of the magnetic head movement tracking device. (**B**) Trial structure, timing and visual cues presented to the subjects. The white square indicated the preparation cue, followed by the white arrow indicating the direction of hand (or head) movements to be performed between movement onset (green square) and end (red square). The trial number was displayed continuously in the lower left corner of the screen (numbers not in scale).

Head movements were recorded with the magnetic tracking system ‘Patriot’ from Polhemus. The device recorded the position and orientation (six degrees of freedom) with a resolution of 11.7 µm and 0.0031°, respectively, for source-sensor distances around 30 cm. The sensor was attached at a medial occipital position at the fNIRS helmet. The source creating the magnetic field was placed at approximately the same height and 30 cm behind the sensor (Figure 1A). The head position was sampled at 30 Hz. The smallest distance between the magnetic sensor and the metal sheaths of all optodes was approximately 6 cm. We could not detect any differences in the measurements of the tracking system with or without the helmet and optodes (similar precision according to visual interpretation of data recorded during extended position tracking).

The tracking system also provided a stylus, which was used to determine the 3D-coordinates of the optodes and the surface of the scalp.

Control of the tracking system and the presentation of visual cues were realized in separate Matlab programs. Both Matlab programs as well as the software controlling the fNIRS system ran on the same computer, which allowed for synchronization of the three systems based on system time.

### Experimental Setup and Paradigm

**Figure 2:**
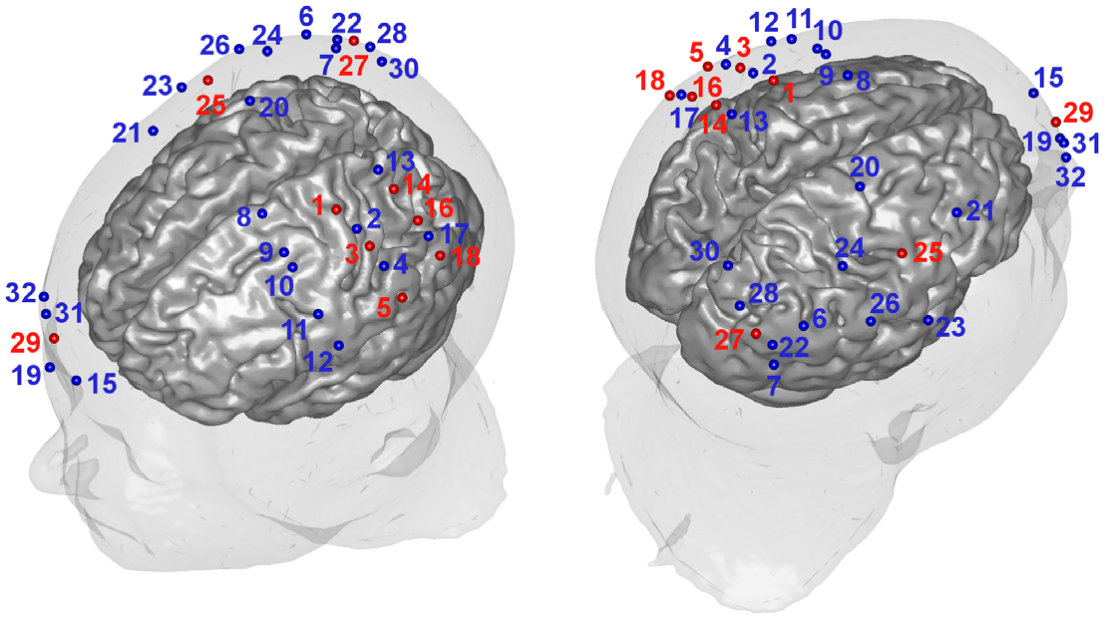
Optode setup, numbers and positions mapped on the scalp (red - source, blue - detector).

Subjects were seated approximately 60 cm in front of a computer screen and asked to rest their head on a chin rest in order to reduce head movements and support the weight of the fNIRS helmet with optodes during the experiment. Optodes were positioned above contra- and ipsilateral sensorimotor areas (C3 and C4 position, 10–20 system) as well as above ipsilateral prefrontal and occipital areas (Figure 2). The right forearm rested on a pillow to prevent arm and shoulder movements (Figure 1A). The posture was adjusted so that the right hand was relaxed and could move without contact to objects or obstacles.

Subjects continuously gazed at a fixation cross. Visual cues (size ∼1.2°) were presented on the screen according to the sequence shown in Figure 1B. Each trial started (white square) with the hand hanging down in a relaxed position (home position). Subjects were instructed to perform periodic (∼0.25 Hz) hand movements causing the finger tips to alternate between the outer and home position for ten seconds in each trial (directions: left – radial deviation of the wrist and finger extension, up - dorsiflexion of the wrist and finger extension, right - ulnar radiation of the wrist and finger extension, down - palmar flexion of the wrist and finger flexion). Movement amplitudes (∼5 cm) and speeds were approximately the same for all directions. Directions were indicated in a pseudo-random order by a visual cue. A temporal jitter between the preparation (white square) and the directional cue (arrow) prevented periodic signals (e.g. heart beat, breathing or Mayer wave) to influence fNIRS signals in a consistent manner.

We performed a pilot experiment (five subjects) using all four movement directions to estimate which two of the four directions could be distinguished best from the fNIRS signals. In this pilot experiment, subjects were instructed to prevent any head movements while moving their hand in one of the four directions as instructed by the corresponding cue. Each movement direction had to be performed 20 times. No head tracking data were recorded during the pilot experiment.

Based on the results of the pilot experiment, we selected two movement directions (left- and downwards, see Results) in order to increase the number of trials per movement direction in the main experiment. The main experiment (twelve subjects) consisted of two sessions. Each session comprised 30 trials per direction and recordings of head tracking data. In session 1, we instructed the subjects to prevent any head movements while moving the hand. After a short break, we continued with session 2 (control). In this session, the posture was the same but we instructed the subjects to not move the hand but instead to perform small direction-correlated head movements (left - head shaking left right, down - head nodding up down) in the same periodic pattern as previously for the hand in session 1. The amplitude of head movements was similar for both directions and less than 1 cm (< ca. 3° yaw (left) and pitch (down)).

### Data Analysis – fNIRS

The fNIRS signals were low-pass filtered using a 3rd order Butterworth filter. In most analyses, acausal filtering (zero phase shift) with 0.15 Hz cutoff was applied. As acausal filtering requires knowledge about the signal’s future, it cannot be applied in a real-time BMI. Therefore, we applied causal filtering using a corrected cutoff [22] of 0.12 Hz in the decoding analysis. After filtering, the signal was cut into trials ranging from six seconds before movement onset (MO) to 25 seconds after movement end (ME). All trials were used, i.e. no trials were rejected.

Raw fNIRS signals were converted to relative concentration changes according to [23]: division by baseline, taking of the negative logarithm, integration of absorption coefficients. The average signal over the second preceding MO was used as baseline and the absorption coefficients were obtained by averaging the individual coefficients provided in Wray and colleagues [24] and Prahl [25]. Not all theoretically possible 288 channels (source-detector pairs) provided reasonable signals due to too small or large distances. Therefore, data analysis was performed using channels selected in a two-step procedure: First, channels were selected which across all trials only contained real values for relative concentration changes (complex values can occur due to the logarithm used in conversion from negative values in raw data, negative values being caused by strong noise in weak signals). From this group of channels, only those with source-detector distances between 2.5 and 5.2 cm (long-distance channels) were selected. Signals recorded on channels with shorter source-detector distances are unlikely to have penetrated cortical tissue. However, such channels can be used for artifact assessment and are, therefore, included in some analyses (explicitly indicated). Likewise, contralateral sensorimotor signals with corresponding source-detector distances in the range of 0–2 cm (short-distance channels) were decoded for comparison.

### Data Analysis – Head Tracking

The tracking data were filtered (causal) and cut into trials exactly as the fNIRS signals. These data thus reflect changes of head position and orientation with respect to the position and orientation during the second preceding movement onset. The head tracking data were resampled to 7.94 Hz (the sampling rate of the fNIRS data).

### Topographies

The 3D coordinates of the optodes above contralateral sensorimotor areas were reduced to 2D coordinates using PCA, i.e. contralateral optodes were projected on a “best-fit” (least-square) plane using the two principle components with largest eigenvalues of the 3D coordinates. Using the projected 2D coordinates, the previously selected, contralateral fNIRS channels were positioned midway between their corresponding source and detector. Hemodynamic signals of single channels were plotted at this position.

Furthermore, these positions were used to calculate an interpolated topographical activity map (Matlab function ‘TriScatteredInterp’, linear interpolation). The spatial distribution of the signal-to-noise ratio (difference between the mean signals 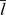 (left) and 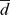 (down) for both movement directions, divided by the standard deviation of the mean corrected trials *l* and *d* across both directions)
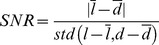
was calculated in this activity map.

### Decoding

The fNIRS and head tracking data were decoded on a single-trial basis with regularized linear discriminant analysis (RLDA; [26]) and non-linear support vector machines (SVM, LibSVM; [27]) with radial basis functions. For each subject, decoding performance of both classifiers were determined as the average across 5–10 times 10-fold cross-validations. The sets of trials used for training and decoding were mutually exclusive. Hyperparameters were determined exclusively using training data. The decoding performance was quantified as the percentage of correctly decoded trials, termed decoding accuracy (DA). As input to the classifier we used the amplitude of the fNIRS signals at single time points (time-resolved decoding) or multiple time points of either single channels, channel groups, all contralateral sensorimotor channels (27 to 42 (Ø35) channels per subject) or all channels above the ipsilateral hemisphere (7 channels for all but one (4 channels) subject). The statistical significance of the individual or average decoding performance for all subjects was estimated using the binomial cumulative distribution of the subject-individual data or the data pooled across all subjects, respectively. With *t* being the number of targets and *n* the number of decoded trials, the probability to predict the correct target at least *k* times by chance is calculated as follows:
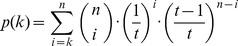
Thus, all decoding accuracies larger than *l = 100 × k/n* are considered significant with a p-value smaller than *p(k)*.

## Results

For the pilot experiment, comprising four movement directions and five subjects, we only calculated the time-resolved decoding accuracy with RLDA (not shown) and found a maximum average accuracy of 36% (binomial test: p<0.01, 3% standard error of the mean (s.e.m.)) around ME. Additionally, we performed pair-wise decoding, i.e. left vs. right, down vs. up, etc. and found that on average across all subjects left vs. down provided a slightly higher decoding accuracy (71%) than pairs of the other directions (58% to 64%). We decided to use left vs. down in the main experiment with twelve additional subjects.

All results presented in the following are based on the main experiment.

### Characteristics of fNIRS Signals Related to Movements of One Hand in Different Directions

#### Comparing signals of the same movement direction

**Figure 3:**
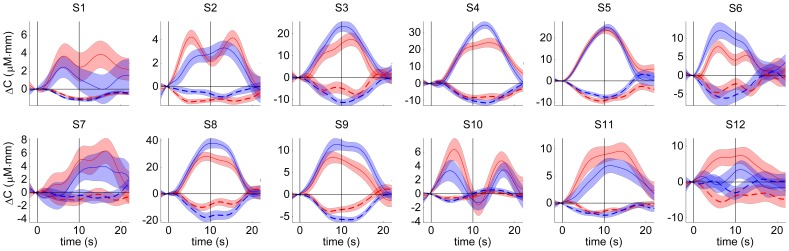
Average hemodynamic responses. Average hemodynamic responses recorded by one exemplary, contralateral sensorimotor channel for each subject (acausal filter, red solid/dashed - HbO/HbR leftward hand movements, blue solid/dashed - HbO/HbR downward hand movements, shaded areas - standard error of the mean, ordinate - concentration change, abscissa - time relative to movement onset, vertical black lines - movement onset and end).

For all subjects and independently of movement direction, several channels above contralateral sensorimotor areas detected movement related hemodynamic responses (Figure 3). Besides this common pattern, the waveform of the signals was not uniform across subjects but differed in characteristics like maximum amplitude, time point of maximum amplitude, and duration to decline to baseline after ME, or also whether the signals were uni- or bimodal.

**Figure 4:**
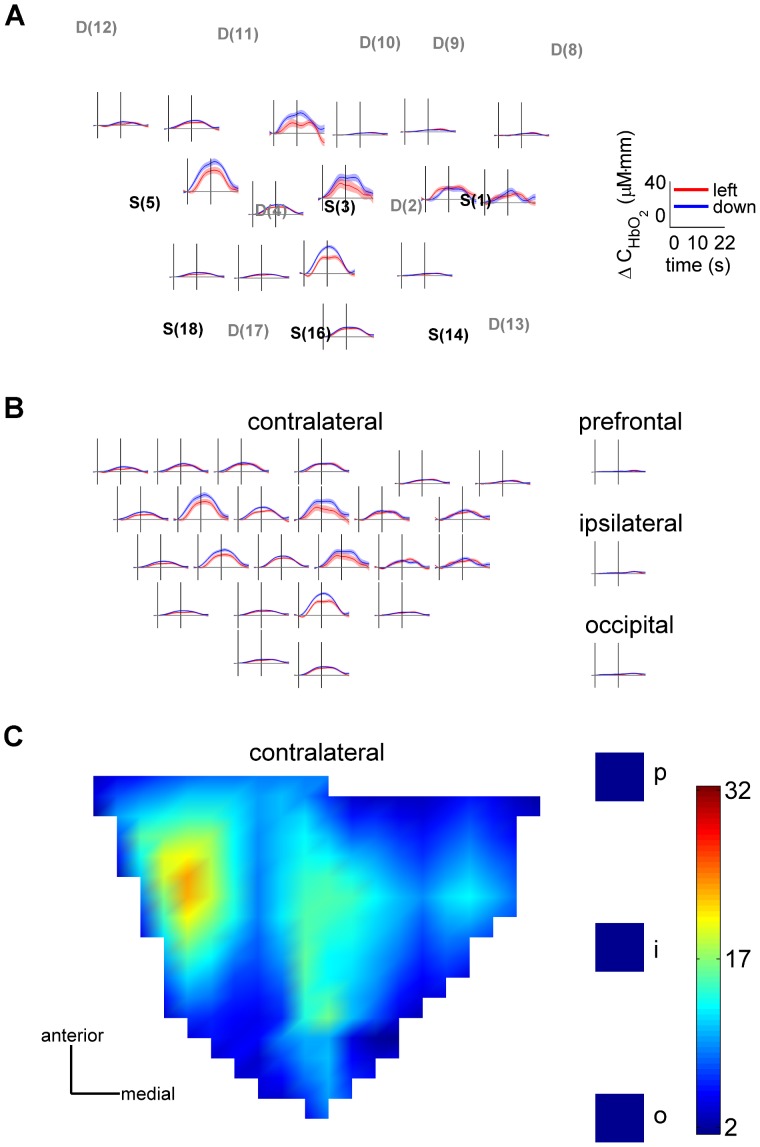
Topographic distribution of hemodynamic responses for subject 8. (**A**) Contralateral sensorimotor optodes mapped on a plane (3D positions shown in Figure 2) and average hemodynamic responses (HbO, acausal filter, Figure 3) for exemplary contralateral sensorimotor channels (insets). Insets are positioned in relation to the sources (S) and detectors (D). (**B**) Same as (A) but hemodynamic responses interpolated using original signals and channel positions. Insets in the right column show the hemodynamic responses averaged across trials and channels above the ipsilateral-prefrontal, ipsilateral-sensorimotor and ipsilateral-occipital brain areas as indicated (ipsilateral channels with source-detector distances <5.2 cm). (**C**) Hemodynamic responses for leftward movements as in (B) but color-coded amplitudes (in µM*mm) around movement end. This presentation is used in Figure 5.

**Figure 5:**
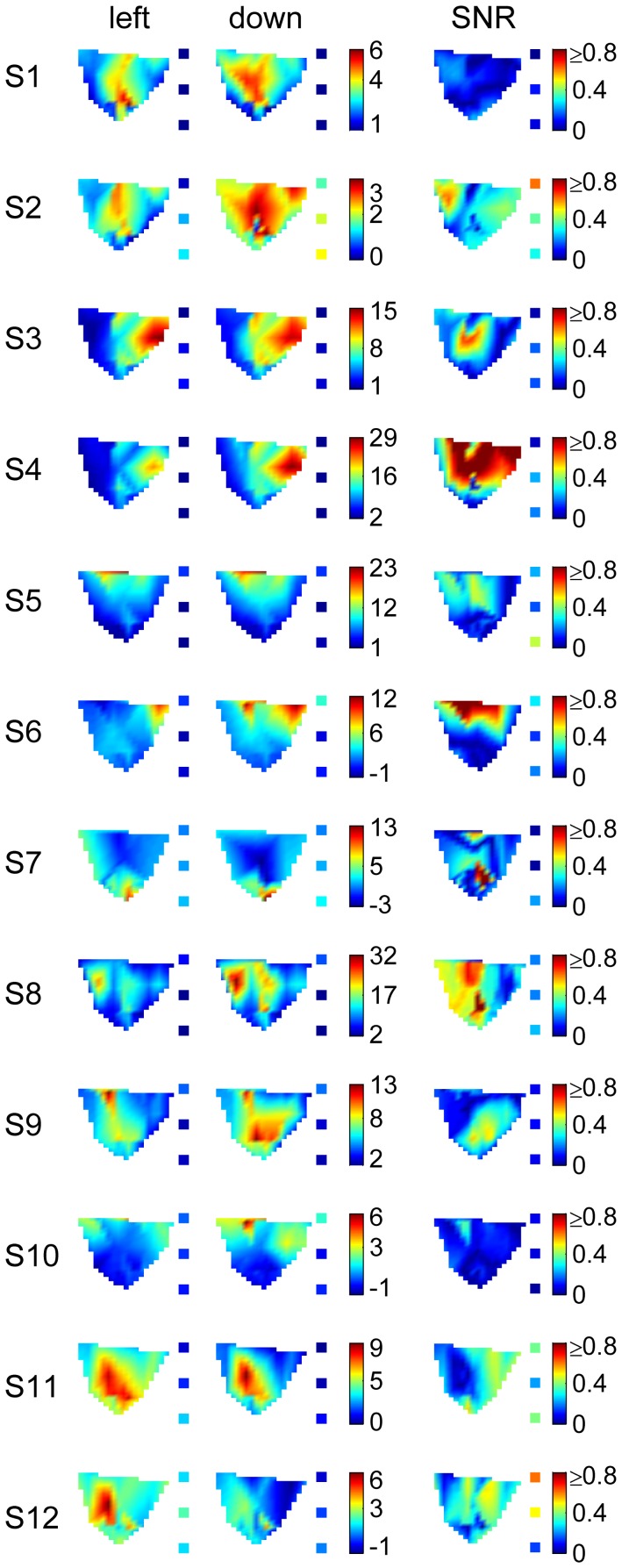
Topographies for each subject around movement end. Left and middle column: Interpolated color-coded amplitudes (in µM*mm) of average hemodynamic response (HbO, acausal filter) for left and downward movements, right column: interpolated signal-to-noise ratios (color-coding equal for all subjects). The small insets adjacent to each topography show the color-coded amplitudes of the hemodynamic responses averaged across trials and channels above ipsilateral-prefrontal, ipsilateral-sensorimotor and ipsilateral-occipital brain areas (from top to down, ipsilateral channels with source-detector distances <5.2 cm).

fNIRS signals measured above contralateral sensorimotor areas were always substantially stronger than those above ipsilateral sensorimotor areas (paired one-tailed t-test: p<0.01 for ten subjects, p<0.08 for two subjects; source-detector distances <5.2 cm). Movement related neuronal activity in ipsilateral sensorimotor areas is known from electrophysiological studies [28]–[32] as well as fNIRS [3], [33]. Furthermore, hemodynamic responses above prefrontal or occipital areas were always lower than those above contralateral sensorimotor areas, comparable to those above ipsilateral sensorimotor areas or not detectable (four subjects) (example subject 8: Figure 4, all subjects overview: Figure 5).

#### Comparing signals for one movement direction against the other

Within subjects, the trial-averaged fNIRS signals for both directions were almost identical for some channels, for other channels the signals differed in amplitude and for some subjects also in waveform (Figure 3). However, there was no systematic amplitude difference across subjects, i.e. for some subjects leftward movements caused higher hemodynamic responses than downward movements and vice versa for other subjects. In five subjects, responses for leftward movements were for some channels higher and for other channels lower than those for downward movements.

#### Topographies

To visualize the topographical distribution of hemodynamic responses elicited by the two movement directions, we computed an interpolated map of the activity measured above contralateral sensorimotor areas and computed the activity above prefrontal, ipsilateral sensorimotor and occipital areas by separately averaging the signals recorded by the corresponding channels (Figure 4). For all subjects, the interpolated activity map of the contralateral sensorimotor areas showed a focal increase of oxygen supply (Figure 5). The location of this focal increase was similar for both movement directions.

Based on the activity map (contralateral sensorimotor area) and the averaged signals (ipsilateral hemisphere), we computed the topographic distribution of SNRs for left- versus downward movements. The SNR maps for individual subjects were heterogeneous (Figure 5). For some subjects, the area of highest SNR was focal and often coincided with the area of strongest activity. For other subjects, this area did not coincide with the area of strongest activity and was not focal, but also never uniformly covered the complete map. For all subjects (except subjects 2 and 12), the SNR of the contralateral sensorimotor areas was higher than that of ipsilateral prefrontal, sensorimotor and occipital areas.

### Decoding of Movement Direction

#### Decoding of fNIRS signals

**Figure 6:**
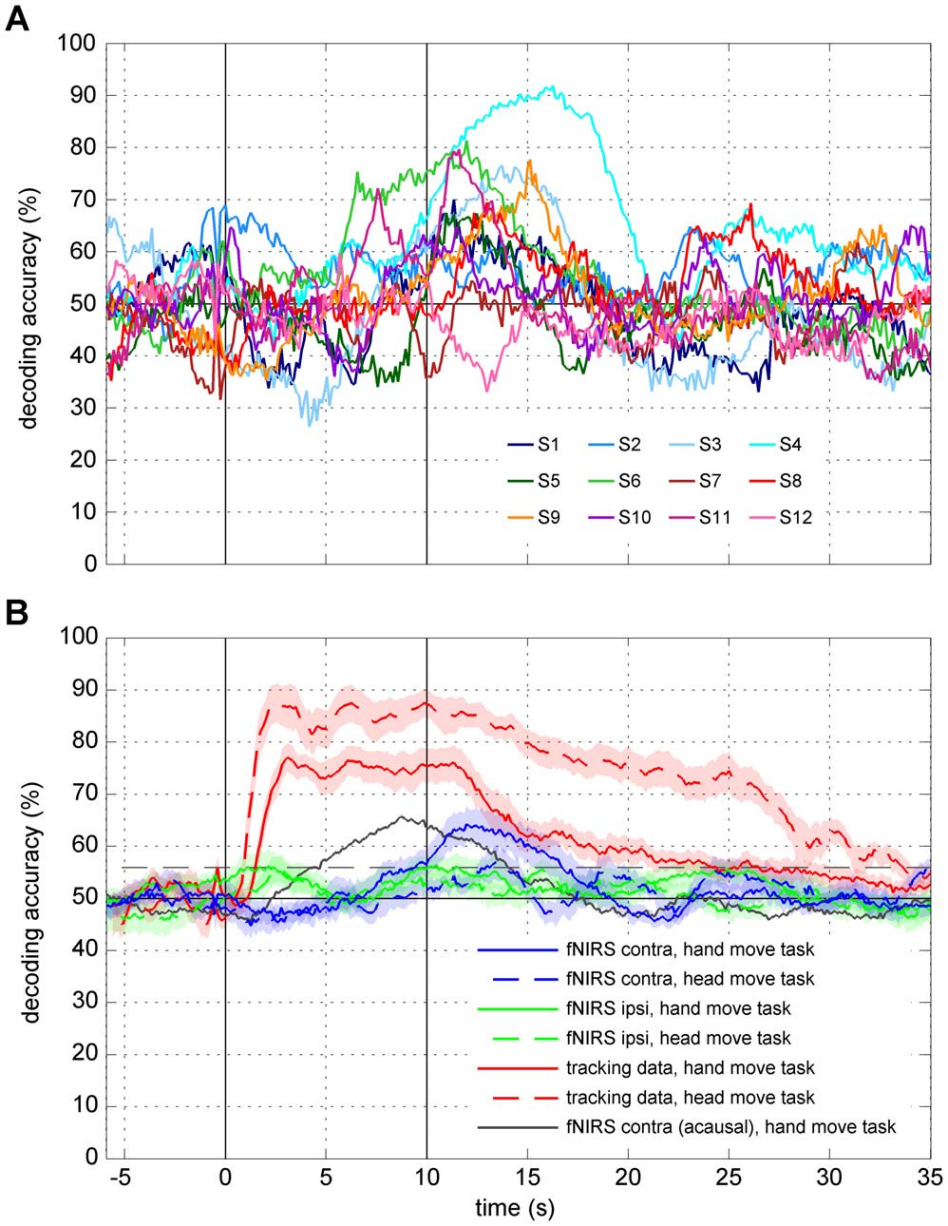
Time-resolved decoding accuracies. Time relative to movement onset, vertical black lines indicate movement onset and end, horizontal black line indicates chance level (50%). (**A**) Subject individual decoding accuracies (DAs) computed using fNIRS signals (HbO, causal filter) recorded above contralateral sensorimotor areas during hand movements (session 1). (**B**) Average DAs computed using precise head tracking data or using fNIRS signals (HbO, causal filter) recorded above different brain areas and during the different tasks (sessions 1 and 2, see Materials and Methods). Time-resolved DA based on acausally filtered fNIRS signals is shown for comparison (gray solid line, see Methods). Shaded areas reflect standard error of the mean and the dashed line the significance level (binomial test: p<0.001) for the average decoding accuracy across all subjects.

Using RLDA to decode single-trial fNIRS signals recorded above contralateral sensorimotor areas during left- and downward movements of the right hand, we found the decoding performance to vary across subjects (Table 1 and Figure 6A).

**Table 1:** Maximum decoding accuracies (DAs) within the time window 5–20 seconds after movement onset.

subject	S1	S2	S3	S4	S5	S6	S7	S8	S9	S10	S11	S12
DA (%)	67*	61	75***	91***	66*	78***	54	68**	74***	62*	79***	54

DA values extracted after 0.5 Hz low-pass filtering the DA curves (Figure 6A) to reduce fluctuations of DA due to noise. Asterisks indicate a significant DA with *p<0.05, **p<0.01 and ***p<0.001 (binomial test, false discovery rate corrected for multiple testing).

For comparison with the head tracking data and to provide a general estimate of what performance to expect when in such a BMI, we focused on the time-resolved decoding curves (Figure 6A) for all following results. Averaging decoding curves across all subjects revealed a maximum DA of 64% (binomial test: p<0.001, 3% s.e.m.) around 12 s after MO (Figure 6B; values for acausal filter: max 66% around 8.7 s, 2% s.e.m.; hence ∼65% as reported in the abstract). DA started to continuously increase from chance level around 7 s after MO, exceeding the significance level (binomial test: p<0.001) around 9.3 s and reaching a plateau around 11.3 s after MO (acausal filter: 2.4, 4.5 and 8.3 s, respectively). DA declined after ME and fell below significance level around 6.9 s later (acausal filter: 4 s).

Importantly, DA for contralateral sensorimotor areas remained around chance level when only channels with source-sensor distances 0–2 cm were used as input to the decoder (not shown). Likewise, the signals measured with channels above the ipsilateral hemisphere yielded a DA which fluctuated around chance level and never reached significance (Figure 6B).

Decoding performance decreased to 58% (causal, 59% acausal) if the HbR instead of HbO signals were classified. Feature vectors composed of both signals did not improve decoding accuracy (HbO+HbR: 65% causal, 64% acausal) compared to the decoding accuracy obtained using HbO alone.

Likewise, non-linear classification or different preprocessing of the fNIRS signals as well as decoding from multiple time points did not improve the decoding accuracy for HbO: using non-linear SVMs with radial basis functions and a grid search for parameter optimization (kernel size and either nu or C), yielded on average across subjects a DA around 63/66% (C/nu, 4/3% s.e.m.; values for acausal filter: 65/64%, 4/3% s.e.m.; all DAs significant with p<0.001, binomial test), similar to the decoding accuracy obtained by RLDA (no significant difference between both SVMs or between either SVM and RLDA, Wilcoxon signed rank test p>>0.05). Using the cutoffs 0.3 or 0.5 Hz for the low-pass filter or a sliding window including multiple time points as input to the RLDA also resulted in similar peak decoding accuracies (63%, 3% s.e.m.; for acausal filter: 66%, 3% s.e.m.; all DAs significant with p<0.001, binomial test) on average across subjects.

**Figure 7:**
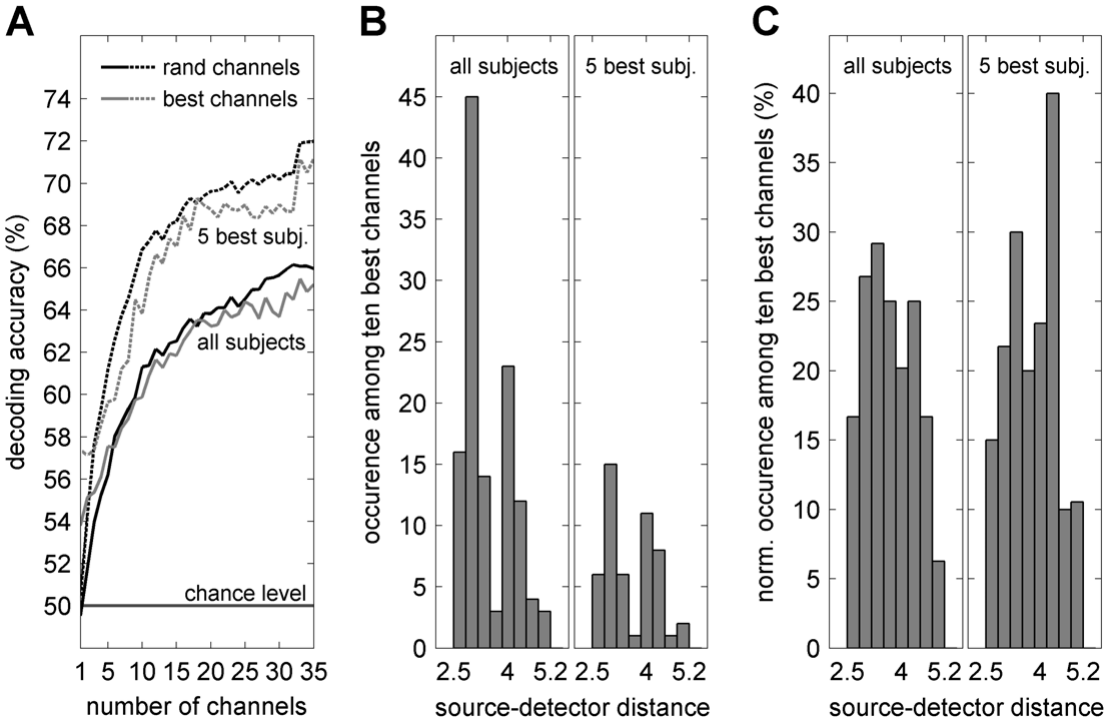
Decoding performance versus number of channels and source-detector distances. (**A**) Decoding accuracy in relation to the number of decoded channels (black – using random channels, gray – using those channels which showed highest (‘best’) decoding on the single channel level, solid – average across all subjects, dashed – average across the five subjects showing highest decoding performance). (**B**) Occurrences of certain source-detector distances among the ten ‘best’ single channels for each subject (left: pooled across all subjects, hence 120 channels in total; right: pooled across the five subjects showing highest individual decoding performance). (**C**) As (B) but normalized by the total number of recorded contralateral channels of the same source-detector distances, yielding the likelihood of a particular source-detector distances to be among the ten channels with highest decoding.

Next, the acausally filtered, contralateral HbO signals at 8.7 s (time point of highest decoding) were decoded using groups of increasing numbers of randomly selected channels, numbers ranging from one to 35 channels. This procedure was repeated 35 times per subject and carried out for the average across all subjects and, due to the high variability over subjects and a possible subgroup of better performing subjects, also separately for the average across the five subjects showing highest decoding performance (Figure 6A and Table 1: S3, S4, S6, S9, S11). The following holds for both groups of subjects: The average decoding performance increases with the number of channels, starting at chance level for one random channel and reaching maximum DA for 35 channels (Figure 7A, black curves). If the group of channels contained only those channels which showed highest single channel decoding (‘best channels first’, single channel DA determined in a ten times 10-fold cross-validation), the decoding performance curves are very similar but start at a higher DA (Figure 7A, gray curves).

Among the ten ‘best’ channels (showing highest single channel decoding) for each subject, most had a source-detector distance around 3 cm (Figure 7B, pooled across all subjects, hence 120 ‘best’ channels in total, or pooled across five subjects, hence 50 ‘best’ channels in total). However, the total number of recorded channels varied strongly across source-detector distances. If the number of ‘best’ channels for each source-detector distance is normalized by the total number of recorded channels for this source-detector distance, the distribution of ‘best’ channels across distances between 2.5 and 5.2 cm is more uniform (Figure 7C). Still, channels with distances between 3 and 4.5 cm tended to be more likely among the ‘best’ channels.

#### Decoding of head tracking data, comparison of sessions 1 and 2

Although subjects were instructed to prevent head movements in session 1 (hand but no head movements), data of the high-precision head tracking system could be decoded with a peak DA of around 77% (binomial test: p<0.001, 4% s.e.m.) on average across subjects (Figure 6B). DA rapidly increased after MO and remained at a plateau until ME. Thus, subjects made unconscious head movements that were correlated to the direction of hand movements and clearly measurable with the head tracker. Yet, the decoding of the head movements yielded a time course of DA which was strikingly different from the time course of the DA of the fNIRS signals, which resembled the time course of the hemodynamic responses (Figure 6B, DA; Figure 3, hemodynamic response).

In session 2 (control: voluntary, small direction-correlated head but no hand movements), decoding of the head tracking signals resulted in a similar time course of DA as for session 1 with an even higher plateau of around 88% (binomial test: p<0.001, 4% s.e.m., Figure 6B). This indicates at least equally strong head movements in sessions 1 and 2, which we further confirmed by comparing the amplitude of head movements of both sessions (amplitudes in session 2 were two to twenty times larger than and rarely similar to those in session 1). Although the DA of the head tracking data was higher in session 2, the DA for the contralateral fNIRS signals from this session fluctuated around chance level.

## Discussion

We showed that hemodynamic signals recorded with fNIRS above contralateral sensorimotor areas vary with the direction of hand movements and can be decoded with ∼65% accuracy. This finding closes a gap in previous research about directional tuning by completing the spectrum of signal types, so far comprising intra- and extracranial electrophysiological recordings and fMRI. The modulation of fNIRS signals by movement direction is weak and the decodable directional information too low for an application in practical BMIs. Our results also demonstrate that fNIRS is relatively resistant to head movements, which makes an application of fNIRS in motor control studies promising.

### Characteristics of fNIRS Signals & Topographies

Across subjects neither the waveform of fNIRS signals nor their directional tuning was uniform, e.g. for some channels the signal amplitude differed with movement direction, for other channels the signal waveform varied with direction (Figure 3). Such signal diversity is a common finding for all recording techniques and implies that also decoders for fNIRS BMIs should be individually trained for each subject to increase performance.

The topographic representations revealed a focal increase of hemodynamic activity in contralateral sensorimotor areas (Figure 5). As the location of this increase was similar for both movement directions, it might reflect movement related neuronal activity in the hand area of the primary motor cortex. Differences in the location across subjects are explained by differences in the positions of the optodes relative to the hand area and by different activation patterns. The area of highest SNR was also focal and often coincided with the area of strongest activity (Figure 5). The SNR of ipsilateral areas was lower than that of contralateral sensorimotor areas.

These findings demonstrate that the strongest signal modulation and most pronounced differences in the signals between both movement directions were found above contralateral sensorimotor areas.

### Decoding of fNIRS Signals

Our results show that on average the directional information of fNIRS is low. Different decoding strategies were applied but could not increase accuracies. The observed differences in decoding performance across subjects (Figure 6A, Table 1) could be due to a suboptimal optode placement in some subjects (e.g. subjects 5, 7, 10, Figure 5) but also indicate that for some subjects (e.g. S4 and S6, Figure 6A) fNIRS can allow for higher decoding performance. At this stage it is unclear whether there are two groups of subjects, one with directional fNIRS tuning and the other one without; the maximum DAs did not indicate any multi-modality but rather a broad distribution of decoding performance. A higher number of subjects would allow investigating a potential grouping of subjects according to their decoding performance.

Using HbR instead of HbO signals resulted in a performance decrease. Thus, the HbR is less informative with respect to hand movement direction (unilateral), which is in contrast to left hand versus right hand movements, for which HbR can allow for similar decoding performance as HbO [3] because control of these movements is spatially separated into the motor areas of the two hemispheres.

Compared to electrophysiological recordings ranging from single- and multi-unit activity over local-field potentials and electrocortico- to electroencephalo- and magnetocencephalograms (for a comparison see [16]), fNIRS allowed for a much lower accuracy in decoding hand/arm movement direction. This low performance for fNIRS might be caused by the low spatial resolution and extra-cortical sources (e.g. movement-unrelated changes of blood flow in the scalp), which influence the absorption of near-infrared light. In a related fNIRS study, Sato and colleagues [34] estimated the direction of high isometric forces (15 N) applied with the arm in different directions and reported DAs of 87.5% for two and 55.5% for four directions. A comparison is problematic as that study used large isometric forces and did not provide information about the recorded signals.

Is it possible to increase the directional information of fNIRS by using a higher resolution optode arrangement? We used six sources and 15 detectors over the contralateral sensorimotor area, which is comparable to previous studies on decoding fNIRS signals from sensorimotor areas [3], [10]–[12]. A more dense arrangement of optodes might increase the decodable information; however, source-detector distances below approximately 2 cm are unlikely to provide reliable signals from cortical sources. Our results show indeed that channels with source-detector distances between 3 and 4.5 cm are more likely to contain high directional information compared to channels with shorter (2–3 cm) or longer (4.5–5.2 cm) distances (Figure 7C). As the density in the used optode arrangement was already high, adding further optodes would mainly yield additional short-distance channels and is therefore unlikely to substantially improve accuracy. However, performance might benefit from an optimized arrangement of optodes which could for instance be obtained by an additional calibration procedure or by localizing the hand area in primary sensorimotor areas by fMRI. Whether any of the above means can boost the accuracy of directional decoding such that it can actually be used in a practical BMI remains a question to be addressed in future studies.

### Decoding of Head Tracking Data

Head movements could be decoded from head tracking data in both sessions (Figure 6B). If the DA obtained for the contralateral fNIRS signals were based on head movement artifacts, we would, due to identical data processing, expect (a) a similar time course of DA of fNIRS and head movement data (increase immediately after MO, plateau from MO to ME) and (b) a DA significantly above chance for session 2 (head movements but no hand movements). We did not observe these effects. Even in the presence of head movements, the fNIRS signals of contralateral sensorimotor areas did only carry information about movement direction when hand movements were performed (session 1). Further tests (not shown) revealed that head movements of large amplitudes (≈>10 cm) do affect fNIRS due to mechanical displacement of optodes.

### How can fNIRS Signals Vary with the Direction of Hand Movements?

Task-related changes in fNIRS signals could originate from extra-cortical factors such as task-related displacement of optodes or changes in skin blood flow [35]. Whereas movement artifacts would affect both hemispheres and channels independently of source-detector distances, changes in skin blood flow should affect short-distance as well as long-distance channels. Furthermore, if head movements caused changes in skin blood flow, these changes should also occur in session 2 (no hand but only head movements) and would be most likely bilateral and wide-spread. However, we observed: significant decoding only for long-distance contralateral sensorimotor channels, no decoding above chance for short-distance contralateral sensorimotor channels or for signals from the ipsilateral hemisphere, focal SNR increases and decoding above chance only if hand movements were performed. Together with the head tracking controls, our findings, therefore, show that the fNIRS signals reflected tuning of cortical, hemodynamic responses related to hand movements.

Tuning of neuronal population activity to movement parameters has been demonstrated repeatedly [31], [36]–[39], yet its origin especially in the context of extra-cranial recordings is not understood. In motor tasks, the intensity of hemodynamic responses, recorded with fNIRS, seems to be directly related to the force [10], [40], [41] and complexity [9] of movements, whereas the relation to movement frequency seems to be less direct [42], [43]. As we did not find a direct relation between the hemodynamic response and the movement direction (e.g. leftward movements do not uniformly cause higher responses) and as we instructed the subjects to perform both movements with the same frequency, our findings indicate that none of these three parameters (force, complexity, frequency) had a prominent influence on the measured fNIRS signals.

Directional tuning in recordings reflecting the activity of large cortical areas might be explained by a large-scale “muscle map” because different movement directions require different muscles or muscle activation patterns. However, such a map has not been found and intra-cortical stimulation studies in monkeys do not provide evidence for the representation of muscles in distinct, separate areas [44].

Instead, previous studies suggested that directional tuning in 3×3×3 mm fMRI voxels is observed due to neuronal clusters with similar preferred directions [20]. It has been suggested [20] that these clusters reflect mini-columns composed of neurons with similar preferred directions as found in single-unit recordings in monkey [45]. Given these findings one might explain the tuning of the lower resolution fNIRS by assuming that fNIRS reflects the activity of multiple fMRI voxels. To investigate whether this can explain the strength of directional tuning found here would require (1) a quantitative assessment of the resolution of fNIRS as well as its signal-to-noise ratio given the influence of extra-cortical confounding signals, and (2) assessing the distribution of directional preferences over the range of fMRI voxels presumably underlying the coarser fNIRS signal.

Alternatively, it has been shown that directional tuning of neuronal population signals can emerge even in the absence of any organization of preferred directions ([16]; see also [46] for a similar case in the primary visual cortex).

In summary, our findings demonstrate that fNIRS allows for investigating cortical activity associated with unilateral hand movements. The signals vary with movement direction but directional information is too low to be used as a control signal in practical BMI applications. Utilization of fNRIS in motor control studies seems particularly promising as we found fNIRS to be relatively resistant to head movements.
